# A decoupled circuital model methodology for calculating DC currents in AC grids induced by HVDC grounding current

**DOI:** 10.1371/journal.pone.0209548

**Published:** 2019-01-10

**Authors:** Lingyu Zhu, Chenzhao Fu, Hao Liu, Dandan Zhao, Lei Su, Habibur Rehman, Shengchang Ji

**Affiliations:** 1 Xi’an Jiaotong University, Xi’an, Shaanxi, China; 2 Electric Power Research Institute of Shanghai Electric Power Company, Shanghai, China; 3 State Grid Chengdu Power Supply Company, Chengdu, China; 4 American University of Sharjah, Sharjah, UAE; Institute of Materials Science, GERMANY

## Abstract

Large currents are injected into the earth from grounding poles of HVDC systems under monopole ground return mode. The currents change the earth surface potential and result in DC currents in AC systems. This paper proposes a computationally efficient decoupled circuital calculation method for assessing the unwanted DC currents in AC grids. Firstly, the earth resistive network is acquired by simulating the DC grounding current distribution using Finite Element Method (FEM). Secondly, the earth resistive network and AC grid are combined to develop a decoupled circuital model of the overall system. The acquired model is used to calculate the DC currents in AC grids by solving a set of linear equations. The proposed method is computationally more efficient as compared to field-circuit coupled methods. In addition, its accuracy is proved by showing a close agreement between our results and field-circuit coupled model as well as the actual measurements. Finally, in Shanghai area power grid the DC currents are calculated using the proposed technique. Based on these calculations, remedial measures for reducing the DC currents in AC grid are suggested. Our research results indicate that DC currents in AC systems can be reduced by operating the two HVDC projects with opposite polarities.

## Introduction

HVDC system operating with monopole ground return mode injects large currents into the earth causing variations in the earth surface potential, which results in DC currents in the nearby AC power grids [1–7]. There can be several HVDC terminals in a very dense power grid area like Shanghai, and the distances between HVDC grounding electrodes and AC substations are not too large. This can result in high DC current in AC system. The unwanted DC currents are very harmful for the AC grids and should be mitigated. Especially in transformers, these DC currents can produce vibrations, noise, additional core losses, etc. [7–13]. The DC currents mitigation techniques, such as inserting resistors or capacitors between transformer neutral and grounding grid have been proposed in the literature [5, 9–10]. However, such DC current reduction techniques in one substation will tend to increase the DC current in other nearby substations. Therefore, efficient techniques for the comprehensive assessment of the DC currents in the entire AC grid needs to be investigated.

The DC current distribution analysis for different type of grounding poles [1,14–19], the distribution of earth surface potential [1,20–22] and factors influencing the DC currents in AC system [5,7–12] have been studied in the literature. The DC currents calculation in AC systems is a field-circuit coupled problem. This is because the transformer winding and transmission line resistances influence the DC grounding current field distribution in the earth. The moment method [14–17, 20], and finite element method (FEM) [17, 22] were introduced as direct methods for solving the field-circuit coupled problem. These methods are computationally intensive and time-consuming, especially for large power network like Shanghai. Thus, indirect methods using Thevenin theorem [1–2, 23–24] are investigated to reduce the calculation requirement by transforming the field-circuit coupled problem into a resistive network problem. The mutual resistances are neglected in [2], which affect the calculations accuracy. The authors in [1] improved the calculations’ accuracy by incorporating the mutual resistances that are obtained using complex image method. The load flow data are used in [23–24] to obtain the real-time grid information, which is also an effective and accurate method for the DC current calculations in AC system. The direct field-circuit coupled methods [14–17, 20, 22] though are accurate but time consuming, while the indirect methods [1–2, 23–24] are either less accurate [2] or are only suitable for limited soil structures [1, 23, 24]. This motivated us to explore indirect technique which is accurate, not specific for particular land area and yet not computation intensive.

This paper proposes a decoupled circuital method which is not for a specific earth structure and is computationally efficient without compromising its accuracy. Firstly, the ground resistive network is obtained by employing FEM. Unlike the field-circuit coupled method, the FEM is only utilized to calculate the earth resistive network and the coupled problem is transformed into linear circuital problem thus making it computationally less intensive. Secondly, the mutual resistances are included in the ground resistive network obtained from the FEM calculations and solved for DC currents in AC grid using a set of linear equations. This avoids the employment of complex image method making it applicable for any soil conditions.

The paper is organized as follows: Section 2 outlines the problem of DC currents in AC grids under monopole ground return mode and Section 3 presents our proposed circuital model methodology for calculating the DC currents in AC grids. The proposed methodology is applied to Shanghai area power grid in Section 4 and its computational efficiency and accuracy are evaluated by comparing the calculated results with the actual measurements performed in two of the Shanghai area substations. Also, based on these calculations, measures for DC currents mitigation in AC grid are suggested in Section 4. Finally, the contributions made by this work and concluding remarks are presented in Section 5 of this paper.

## DC currents in AC grids under the monopole ground return mode

**Fig 1:**
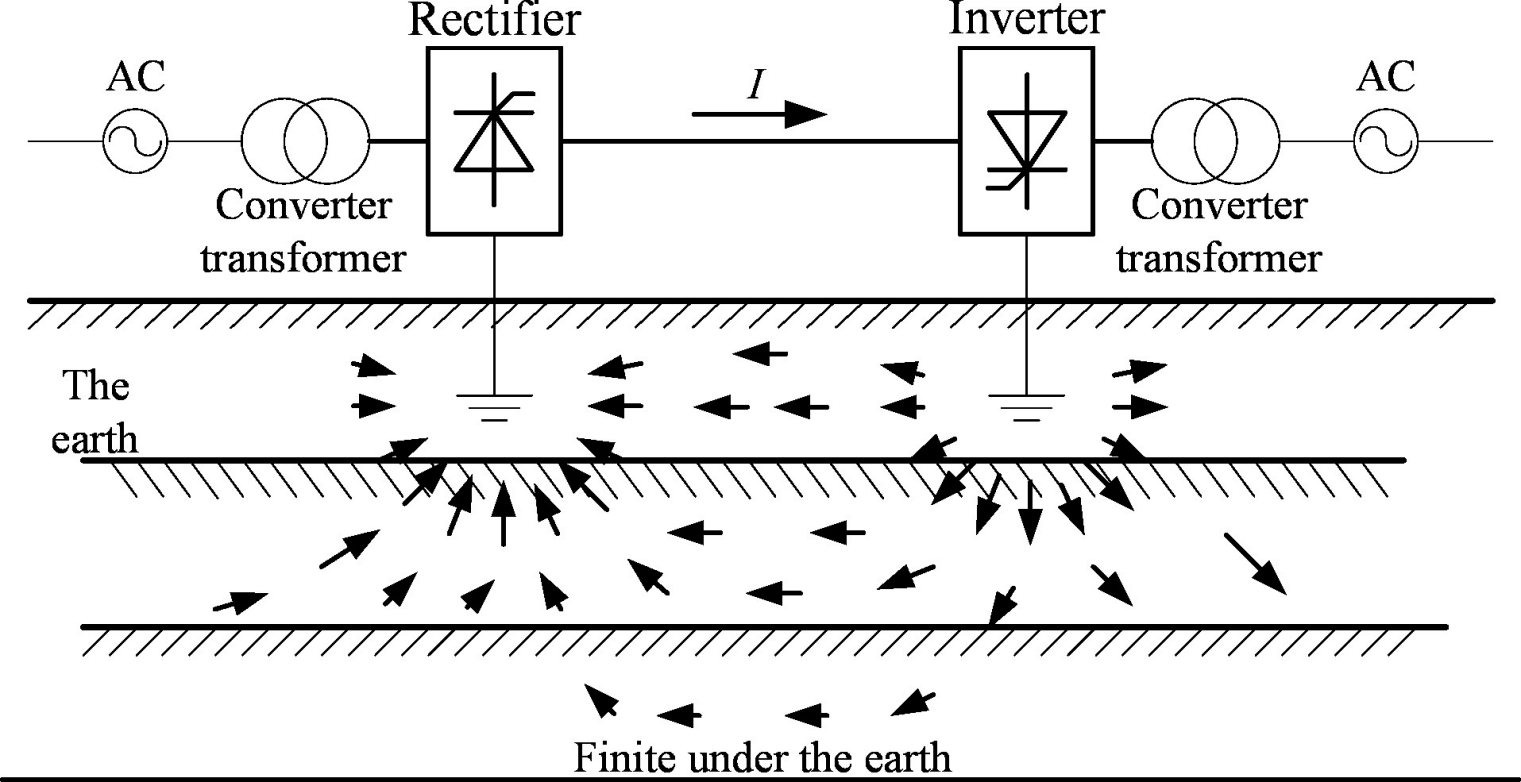
Monopole ground return mode of HVDC projects.

The DC current injected into the earth from grounding pole could be as large as several thousand of amperes when the system is operating under monopole ground return mode caused by system debugging or monopolar failure. In the case of UHVDC, the DC grounding current could reach 4000~4500 A. This large earth current spreads to infinite area as shown in Fig 1 and changes the earth surface potential, which is highly related to the current density distribution in the earth [21].

**Fig 2:**
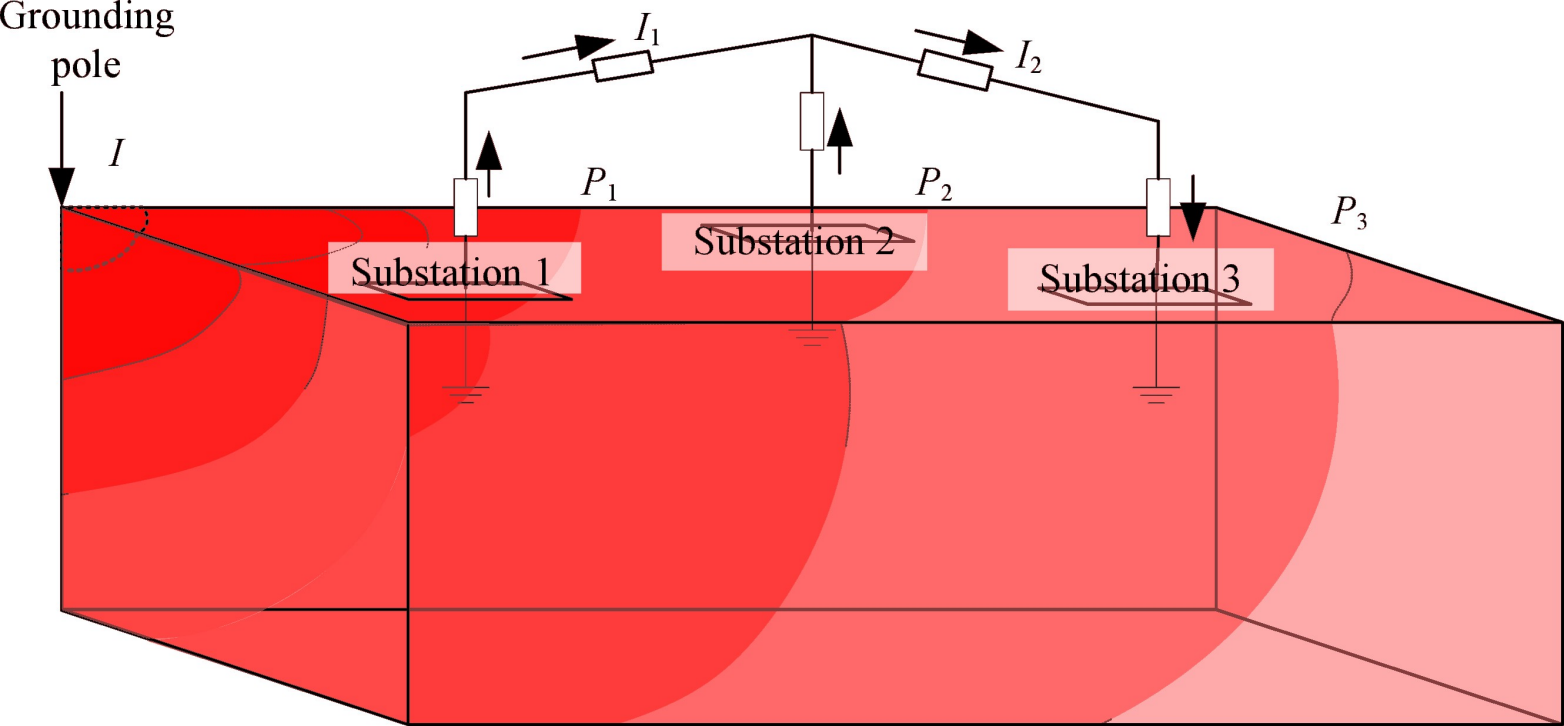
The resistive network above the ground and the electric field under the ground.

It is known that there will be a point to point potential difference on the earth surface when grounding current is injected. Generally, the AC grid substations are located at different earth surface potentials because of the large grounding current in the monopole ground return mode. The potential differences between the substations will cause DC currents in the transformers and the transmission lines between them as shown in Fig 2. These DC currents would be relatively high due to the small DC resistances of transformers and transmission lines. The DC currents are very harmful for the transformers because they can produce additional vibrations, audible noise, core losses, etc. [7–13].

Actually, the current density and the earth surface potential will not only depend on the earth structure but also on the AC grid topology. The transformers and transmission lines resistances develop the connection between different points of the original underground current field and affect the potential differences. This makes DC current calculations in AC grid a field-circuit coupled problem.

## Proposed circuital model methodology for DC currents calculations in AC grid

### Calculation procedure

As discussed in Section 2, the DC grounding current distribution should be analyzed while considering the AC grid connections. This makes it a field-circuit coupled problem. On the other hand, the earth area involved in the calculations should be as large as possible, because the injected grounding current spreads to infinite area of earth. The resistivity of different parts of earth and layers should be taken into account. Furthermore, in numerical field-circuit coupled calculations, the discrete units should be small enough to acquire fine calculation results. However, there is a contradiction between the fineness and the scale of solution because of the limited computation capability, and the sacrifice of either aspect will reduce the accuracy. Therefore, it is necessary to find some alternative DC current calculation method for large areas and complex earth structures.

The earth resistivity of certain part of the earth being an inherent attribute remains fixed and it does not change with or without the AC grid. The resistance of each part of the earth can be acquired by finely and reasonably dividing the earth. The surface potential can be calculated using the acquired circuit model. When AC grid is added in the model, it will be a resistive network model, which can be easily solved. Thus, the field-circuit coupled problem can be transformed to a decoupled circuital problem. The key step is to divide the earth into reasonably small parts and acquire the resistance of each part. This will be discussed in Subsection 3.2.

**Fig 3:**
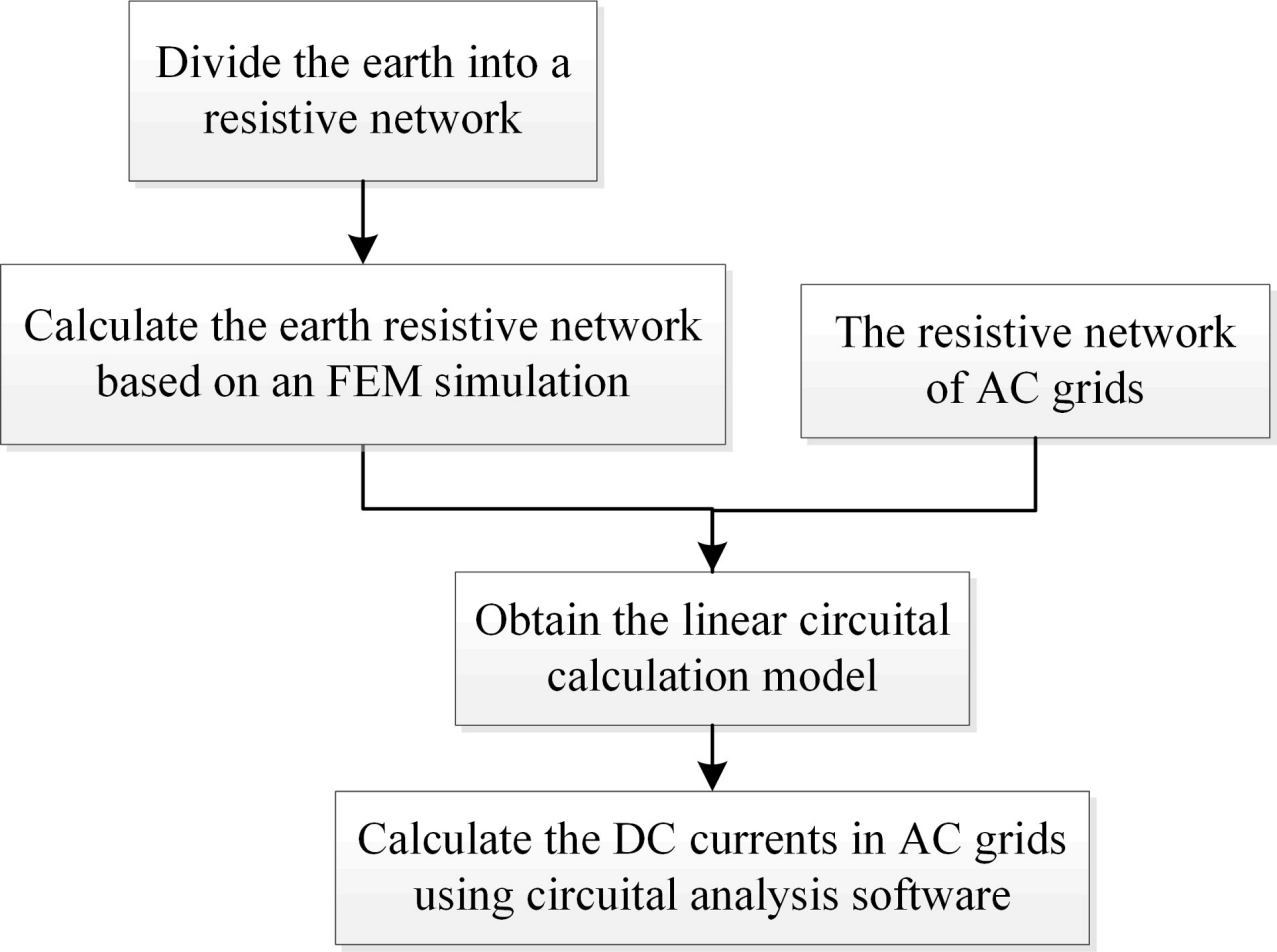
The DC currents calculation algorithm.

The proposed methodology consists of four step processes as outlined in Fig 3. Firstly, the earth is divided into a network of resistors. Secondly, the network resistances are calculated based on an FEM simulation of the grounding current field without the AC grid. Thirdly, the linear circuital model is obtained by adding the AC grid resistances in the corresponding closest node in the earth resistive network. Finally, the DC currents in AC grid are obtained by solving the linear model in a circuital analysis software. The complex circuit model can also be solved by using Lagrange multiplier by minimizing electric energy as discussed in [25]. For the power grid with multiple HVDC terminals, the DC currents in AC grid caused by DC grounding current of each terminal can be calculated separately, and the total DC currents can be obtained by superposition.

### Acquiring earth resistive network

**Fig 4:**
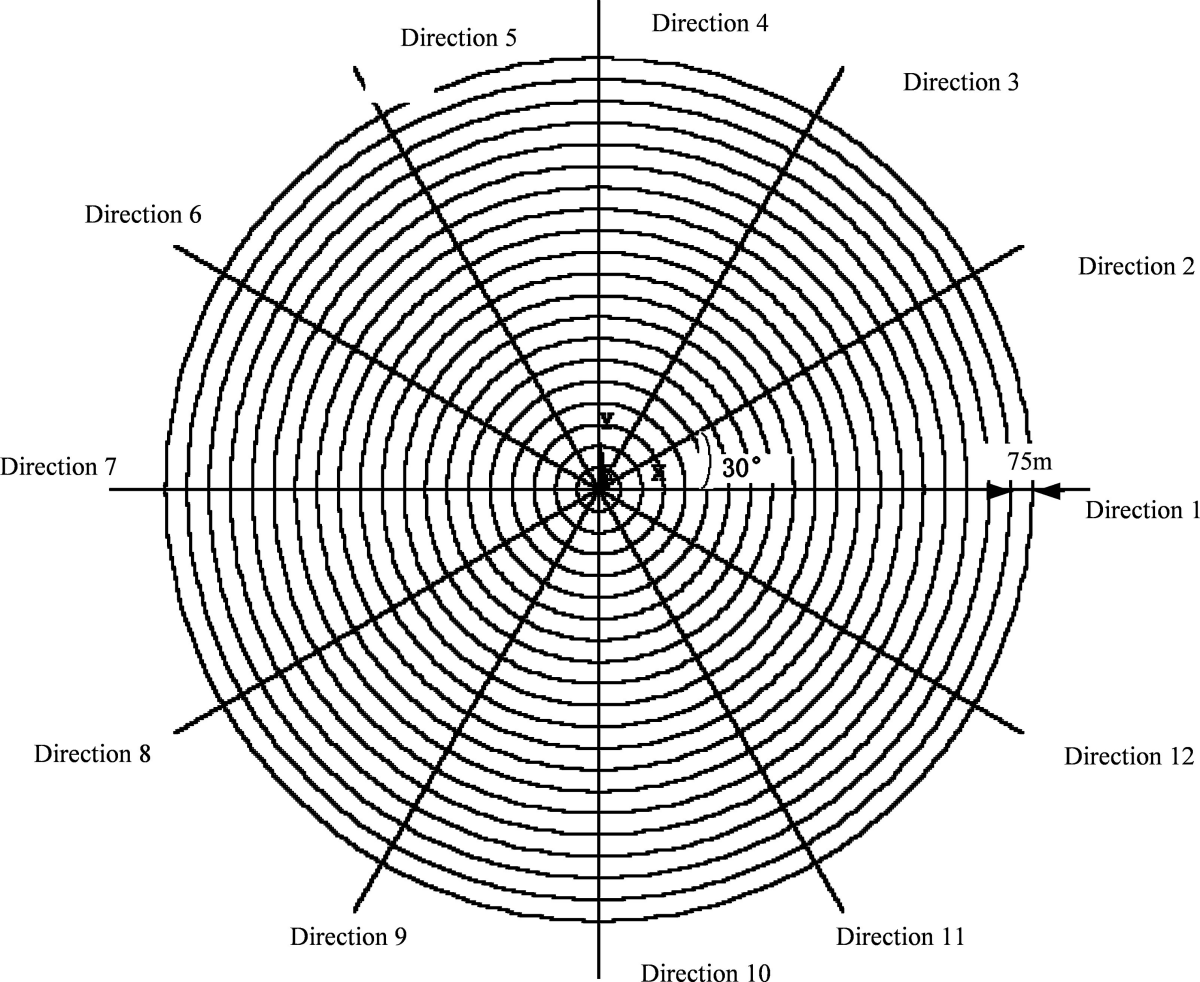
The earth resistive network mesh.

**Fig 5:**
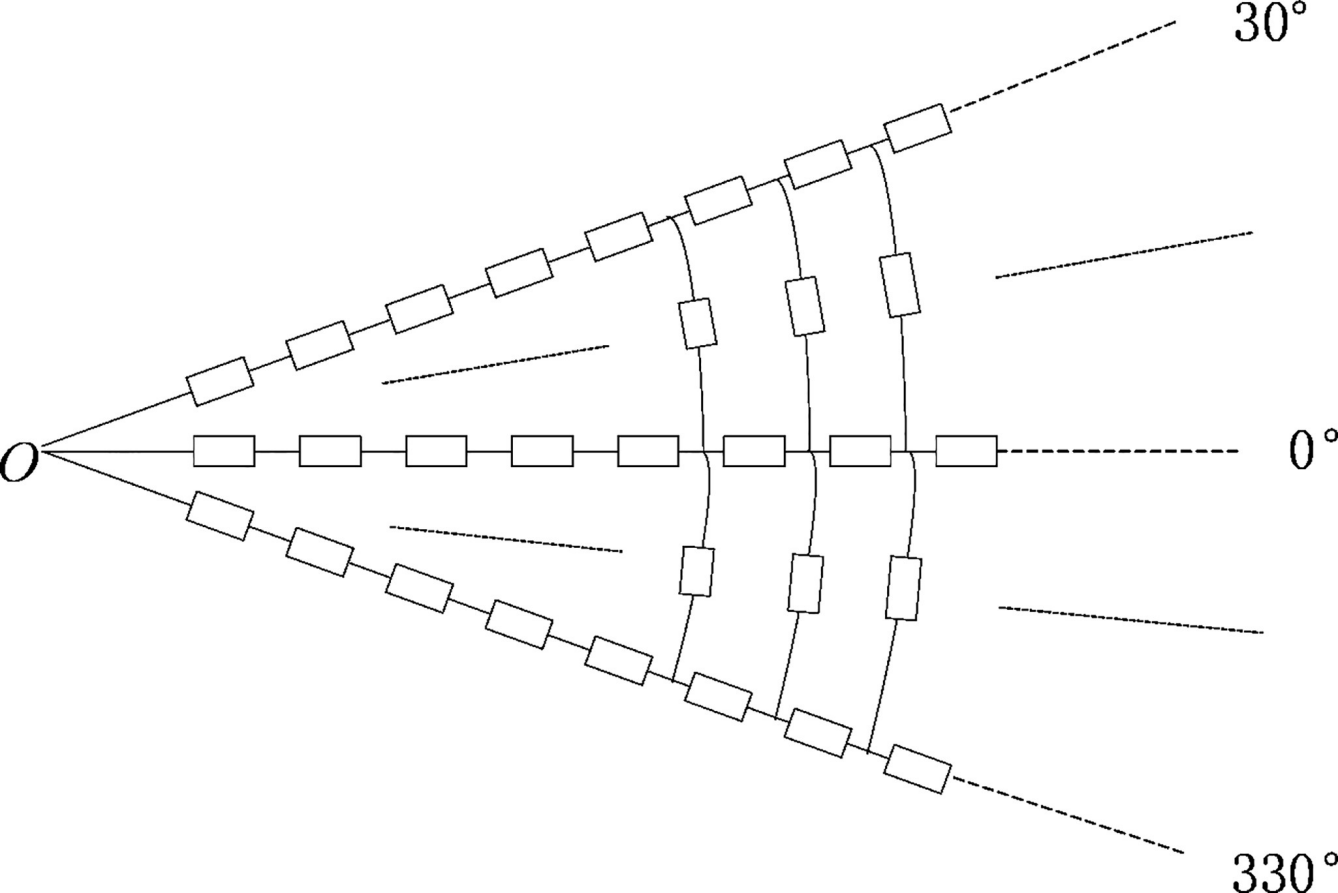
Partial structure of the earth resistive network.

The earth is radially divided into units from the grounding pole [26]. The unit size selection is based on the principle that the discrete resistance network can reflect accurate earth surface potential distribution. Based on a parametric study and comparison with the FEM simulation results, the unit size parameters are chosen as 75 m in the radial direction and 30° in the circular direction, as shown in Fig 4. These units include radial and circular resistors. The largest radius of the calculated model is 150 km, so there are 2000 radial resistors and 2000 circular resistors on or in each radial direction. The radial and circular resistors are connected to each other forming a network as shown in Fig 5.

The soil in each unit can be considered as homogeneous with a resistivity of *ρ*. Therefore, the radial resistance *R*_*r*_ and circular resistance *R*_*θ*_ can be directly calculated by the integral of elemental resistance and conductance respectively, as given by (1) and (2) [26].

$$R_{r}=\rho \int_{r_{1}}^{r_{2}}\frac{dr}{Dr\theta }=\frac{\rho }{D\theta }\ln\frac{r_{2}}{r_{1}}$$ (1)

$$R_{\theta }=\rho \frac{1}{\int_{r_{1}}^{r_{2}}\frac{Ddr}{r\theta }}=\frac{\rho \theta }{D\ln\frac{r_{2}}{r_{1}}}$$ (2)

where, *θ* = 30° which is the circular angle of each unit, *D* represents the depth into the earth whereas *r*_1_ and *r*_2_ represent the unit inner and outer radius respectively. The value of *D* depends on the depth considered which is taken as 15 km in the case study of Shanghai area power grid.

In the case of anisotropic earth, the calculation of the earth resistive network is much more complicated. Hence, an FEM simulation software is used to calculate the DC grounding current distribution and the resistances can be obtained from the quotient of maximum potential difference and average current.

The radial current density and potential of the *k*^th^ element is *J*_*k*_ and *V*_*k*_ respectively. The average radial current of the unit can be calculated by (3).

$$I=\frac{l_{0}}{l}\sum_{k=1}^{n}J_{k}S_{k}$$ (3)

where *l*_0_ is the average length of each element of the FEM model, *l* = 75 m is the unit length and *S*_*k*_ is the average radial area of the *k*^th^ element. The radial potential difference can be obtained by calculating the difference between the maximum potential *P*_max_ and minimum potential *P*_min_ within the unit using (4).

$$V=P_{\max}-P_{\min}$$ (4)

The radial resistance *R*_*ri*_ of the *i*^th^ earth unit can be calculated by (5).

$$R_{ri}=\frac{V_{i}}{I_{i}}$$ (5)

This equation is not suitable for the circular resistance because the circular current and potential difference are usually very small which could result in a large error in the calculation. Therefore, to overcome this problem, the circular resistance is calculated using apparent resistivity *ρ* which can be derived from (1) as;

$$\rho =\frac{R_{r}D\theta }{\ln\frac{r_{2}}{r_{1}}}$$ (6)

Thus, the circular resistance formula can be obtained by substituting (6) into (2).

$$R_{\theta }=\frac{\theta^{2}}{{\left(\ln\frac{r_{2}}{r_{1}}\right)}^{2}}R_{r}$$ (7)

The radial and circular resistances of each unit calculated using (5) and (7) are used to develop the earth resistive network, which enables the DC currents calculation in the AC power grid.

### Influence of grounding electrodes

**Fig 6:**
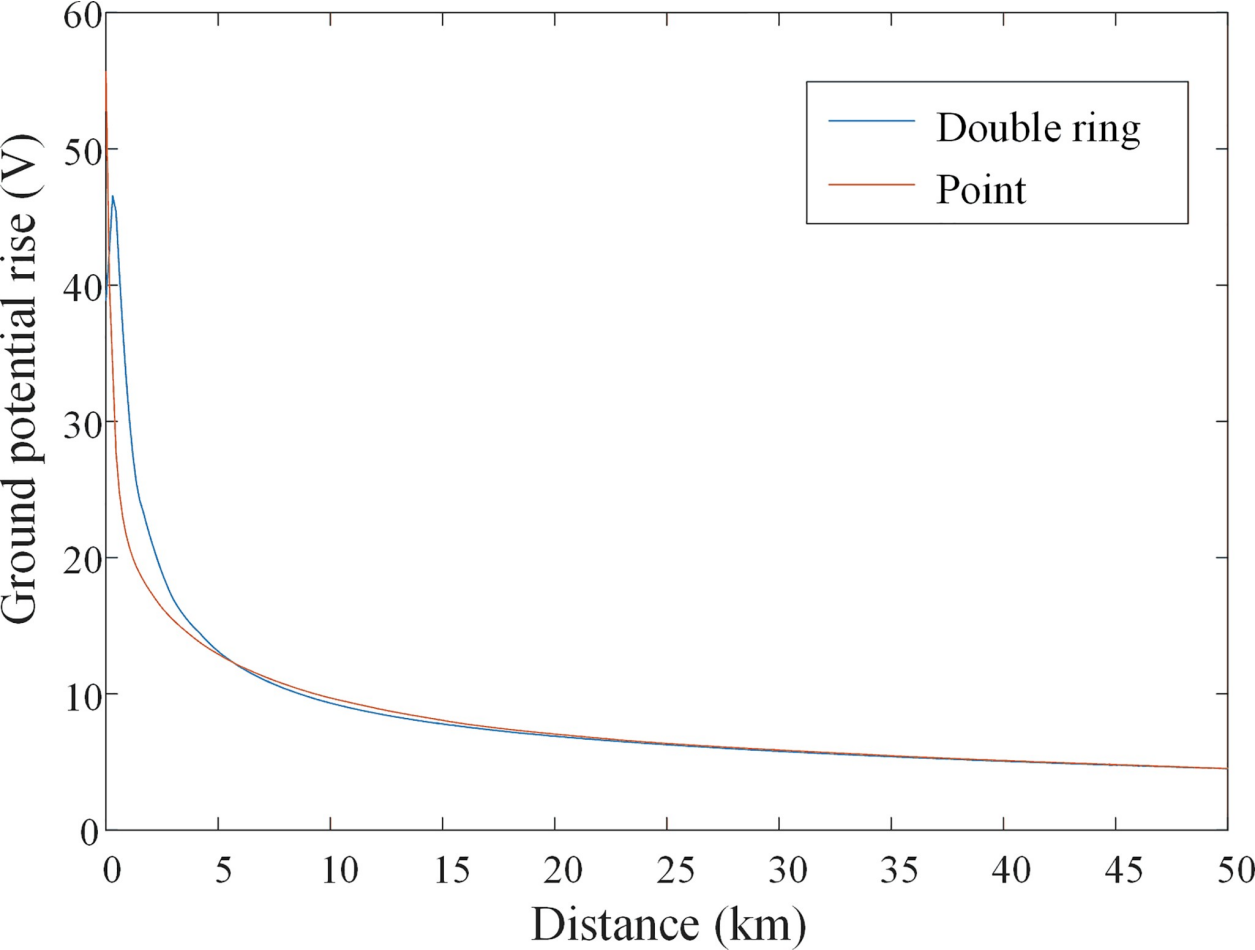
The comparison of ground surface potentials for double ring ground electrodes and point.

A finite element model is built for a double ring grounding electrode to analyze the influence of grounding electrodes on the earth surface potential distribution. The radius of the two rings are 200 m and 400 m respectively, and the rings’ depth is 3 m with a current injection of 4000 A. The same current is also injected for a single point grounding. The simulated potential curves for these two cases along one direction are shown in Fig 6. These plots indicate that resultant potentials are almost the same when the distance is larger than 5 km. The configuration of the grounding electrodes has a significant impact on the total grounding resistance, step voltage, and temperature rise in the soil [26], but not on the surface potentials when the distance is long enough. The distance between grounding electrodes and substations are required to be at least 10 km to reduce the influence of grounding current on the substation operation [26]. Thus, the effect of grounding electrode configuration on the induced DC current can be neglected. Similar conclusion is drawn in [1] as well. On the other hand, if the grounding electrodes configuration is included in the FEM calculation, the meshing near grounding electrodes will be significantly increased and become much more complex. Therefore, in the numerical calculation of the DC currents, it is reasonable to neglect the configuration of the grounding electrodes of HVDC systems.

## Application of decoupled circuital model methodology to Shanghai area power grid

### Shanghai area earth structure

**Fig 7:**
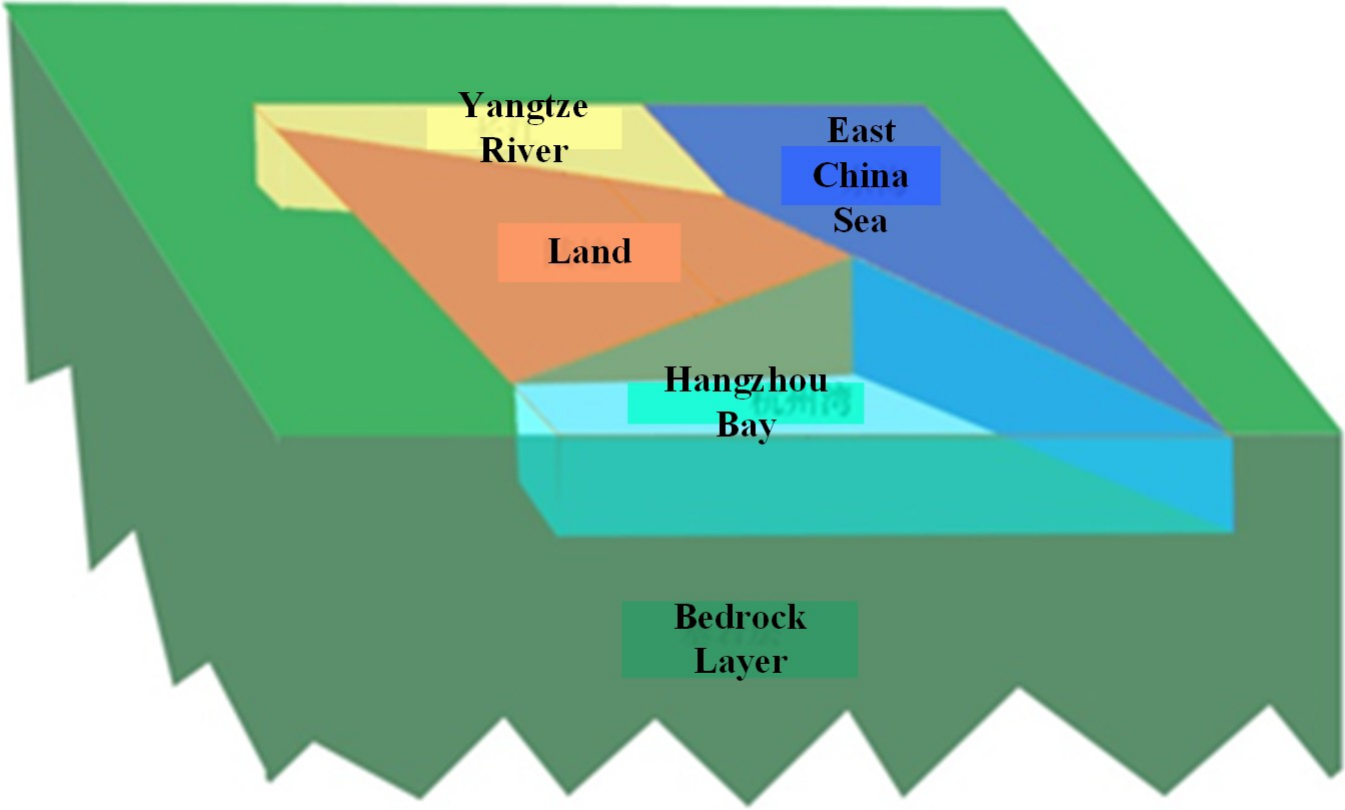
Shanghai area earth structure.

A multi-layer earth model of Shanghai area is built to calculate the DC currents in AC grid. Shanghai area is located at Yangtze River Delta, east side of Asian continent and west bank of the Pacific. It faces the East China Sea to the east and the Hangzhou Bay to the south. It is one of the most developed areas in China and has a very dense power grid. The calculated area is about 400 km × 500 km, and the earth depth being considered is 15 km. As shown in Fig 7, the earth is divided into five parts according to their resistivity, including Yangtze River Basin, East China Sea, Hangzhou Bay, Land and Bedrock Layer. The thickness of the upper layers is 0.35 km while the bedrock layer thickness is 14.65 km. Because of alluvium plain Shanghai area, the resistivity of each part can be considered as uniform, which is consistent with our measured results. The resistivity of each part is shown in Table 1. Moreover, the interfaces are approximated as straight lines to simplify the modelling and calculations.

**Table 1:** Resistivity of different parts of Shanghai area.

Part	Yangtze River Basin	Bedrock Layer	Land	Hangzhou Bay	East China Sea
Resistivity / Ω•m	10	600	10	1	1

**Fig 8:**
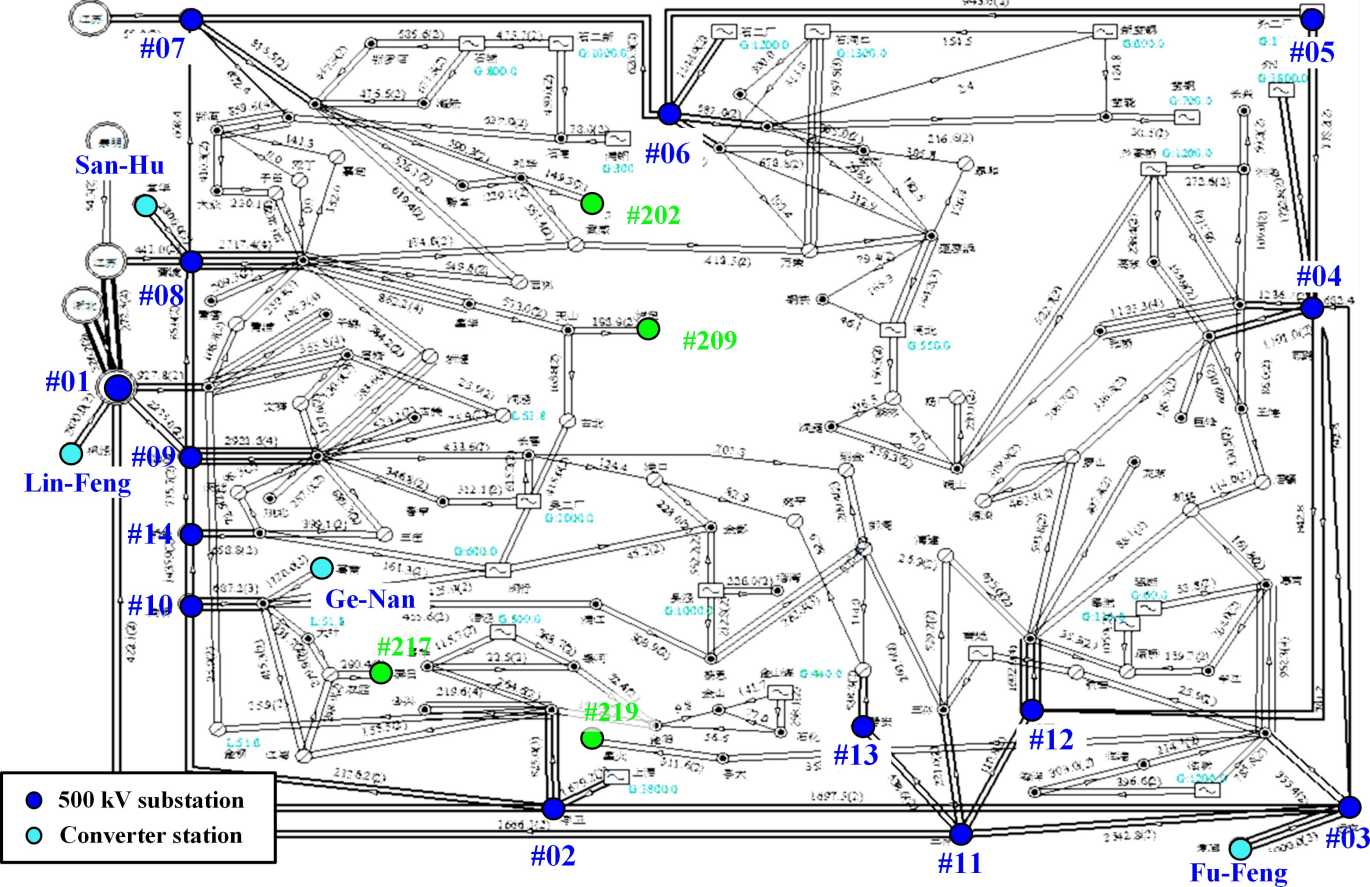
Shanghai area power grid diagram and the positions of the main 500 kV substations and the converter stations.

Four HVDC lines are involved in these calculations, including Ge-Nan HVDC, Lin-Feng HVDC, San-Hu HVDC and Fu-Feng UHVDC projects. The connection diagram of the four converter stations and AC power grids is shown in Fig 8.

Both Ge-Nan and Lin-Feng HVDC projects use Fengjing grounding pole. The transmission capacity of Lin-Feng and Ge-Nan HVDC projects are 3000 MW and 1200 MW respectively. The largest grounding currents of monopole ground return mode are 3000 A and 1200 A for Lin-Feng and Ge-Nan HVDC projects respectively. The grounding pole of San-Hu HVDC project is set at Huaxin and its largest grounding current is 3000 A. The grounding pole of Fu-Feng UHVDC project is set on the boundary of Shanghai and Zhejiang and has a largest grounding current of 4000 A.

Resistances of AC power grids refer to the DC resistances of transformer windings, the neutral point resistances, and the DC resistances of transmission lines. The Shanghai power grid voltage levels are 1000 kV, 500 kV, 220 kV, 110 kV and below. The DC current will not flow into the power grid of 110 kV and below because their neutrals are not grounded. The 1000 kV substations are not taken into account in the calculation because they are distributed very far from each other. Therefore, the problem of DC currents in AC grid primarily needs to be investigated at 500 kV and 220 kV voltage levels. The 500 kV substations are numbered by “#” in sequence as shown in Fig 8, and the 220 kV substations are also represented with numbers in the format of “#2**” in this paper.

### Accuracy and computational efficiency assessment of circuital model methodology

The DC currents in two substations (#02 and #10) of Shanghai power grid were measured by Electric Power Research Institute of Shanghai Electric Power Company. The Fu-Feng HVDC project was operating with monopole ground return mode, and measurements were performed for two grounding currents of 2750 A and 4000 A. The DC currents were measured on the grounding wires of the transformer neutrals by HIOKI “3285 CLAMP ON AC/DC HiTESTER”. This equipment can separate DC current from AC components. The DC current values were directly recorded from the equipment readings working in DC mode. The gross error is 1% or below in the range of 10–200 A, and the manufacturer specifies that measurement error could slightly increase if the current is below 10 A. Geomagnetically Induced Current (GIC) is similar to the neutral DC current induced by large DC current of HVDC grounding electrode and can superimpose on the neutral DC current. The impact of GIC on the measured DC current must be evaluated based on the monitoring of Geomagnetic Disturbance (GMD) activities. There was no GMD report in Shanghai area on the day when the measurements were conducted, which means the GIC impact can be neglected in this data [27].

The DC currents flowing in power grids of Shanghai area were also calculated using both the proposed circuital model and the field-circuit coupled model under the same measurement conditions. The calculated results and average measurement results are shown in Table 2. The comparison shows that proposed decoupled circuital model and field-circuit coupled technique give similar results. Also, the results of both techniques are very close to the actual measurements for the case of substation #2. However, the measured values for substation #10 are slightly lower than the calculated ones. The slight difference between the actual measurements and calculated values can be due to lower accuracy of the measurement device when the current measurements below 10 A as specified by the manufacturer. These results validate that the decoupling and unit sizing has almost no impact on the calculation accuracy which proves the effectiveness of the proposed technique for the DC current calculations of a large area power grid.

**Table 2:** The calculated and measured DC current for 500 kV substation #02 and #10.

Substation No.	Grounding DC current (A)	Proposed method (A)	Coupled method (A)	Measured (A)
#2	2750	-14.6	-14.5	-14.1
	4000	-21.5	-21.4	-20.4
#10	2750	2.0	2.0	1.7
	4000	3.3	3.3	2.9

Next, we compare computational efficiency of the conventional field-circuit coupled method and the proposed decoupled circuital technique. In the conventional approach, the DC currents upon every iteration are obtained using time consuming field-circuit coupled FEM calculations. Our method consists of two step processes as described in Section 3.1. The first step is to acquire the grounding resistive network using FEM calculations. The process of FEM calculation is only performed once. The second step of this process is to solve a linear circuital resistive network which includes the grounding resistive network obtained in the first step and the AC grid resistive network. The computation time for solving the linear circuit is much smaller and therefore can be neglected when compared with the FEM simulation. For example, using a computer with the configuration listed in Table 3, typical calculation time for the first step of the proposed method is 896 minutes (about 15 hours), while the second step requires less than 1 minute. The result of the first step can be directly used in the second step for each iteration performed for different grid configurations as long as the earth model remains unchanged. On the other hand, the computation time for field-circuit coupled FEM method upon each iteration is similar to that of the first step of the proposed method because of the same model size. Therefore, the computational time of the proposed method will be almost (*N*-1) time less than the conventional coupled method as is compared in Table 3, where *N* is the total number of iterations.

**Table 3:** Computer configuration and calculation time.

CPU	Intel Xeon E5-2670
Main frequency	2.6 GHz
Memory	8×8 GB
Proposed method calculation time for *N* iterations	896 min + 1 min× *N*
Field-circuit coupled method calculation time for *N* iterations	896 min × *N*

### Shanghai area DC current calculations in AC grid and mitigation measures

As detailed in section 4.2 the proposed technique for dc current calculation in AC grid requires less computational time without losing the accuracy. In this section we apply the proposed circuital model technique for calculating the DC current in AC power grid of Shanghai area power network and suggest measures for mitigating this unwanted DC current in AC grid. Subsection 4.3.1 investigates the effect of polarity combinations of HVDC lines on the DC currents in AC grid and also recommends the polarity combinations for reducing the DC currents. The AC grid connections is another important factor that influences the DC currents that will flow in AC grid. Therefore, the impact of this factor has been examined in Subsection 4.3.2. Finally, Subsection 4.3.3 analyses the substation location effect on the DC currents in AC grid.

#### Polarity combinations of HVDC lines’ effect on the DC current in AC grid

The voltage polarity of energized pole in monopole ground return mode of an HVDC system is defined as the polarity of monopole ground return system. In this subsection, we have considered the HVDC system effect on 500 kV and 220 kV AC systems for different polarity combinations of Fu-Feng, Lin-Feng and San-Hu HVDC systems while operating in monopole ground return mode. Furthermore, DC current in AC system is calculated with only two HVDC systems energized at a time as shown in Table 4. This is because the case of three HVDC systems operating with monopole ground return mode is quite rare.

**Table 4:** DC currents in main AC substations for various polarity combinations of HVDC lines.

Voltage level	Substation	DC currents (A)					
		Fu-Feng & Ling-Feng		Fu-Feng & San-Hu		Lin-Feng & San-Hu	
		Similar polarity	Opposite polarity	Similar polarity	Opposite polarity	Similar polarity	Opposite polarity
500kV	#03	19.79	8.35	20.48	7.66	12.14	-0.69
	#04	19.80	0.12	19.30	0.62	19.18	0.50
	#05	14.85	-6.41	7.29	1.14	13.70	7.56
	#07	14.61	-1.25	7.98	5.38	9.23	6.63
	#10	-26.31	34.83	7.28	1.23	-27.54	-33.60
	#12	16.04	6.82	12.85	10.01	6.03	3.19
	#13	11.68	-10.00	0.18	1.50	10.18	11.50
	#14	-17.31	-22.30	-21.93	-17.67	0.37	4.62
220kV	#201	5.46	-13.42	-19.03	11.07	-5.61	24.49
	#202	-36.27	-61.17	-53.97	-43.47	7.20	17.70
	#203	-25.21	-21.85	-25.86	-21.20	-4.01	0.65
	#204	-11.43	17.29	3.80	2.06	-13.49	-15.23
	#205	12.90	-14.90	-0.98	-1.01	13.91	13.89
	#206	-11.52	18.96	4.70	2.74	-14.26	-16.22
	#207	-9.86	13.78	3.20	0.72	-10.58	-13.06
	#208	23.66	-13.48	7.28	2.90	20.76	16.38
	#209	-75.16	67.34	-4.93	-2.90	-72.27	-70.24
	#210	-6.86	21.02	8.74	5.43	-12.29	-15.59
	#211	13.89	-10.75	3.22	-0.08	13.97	10.67
	#212	10.17	-0.90	8.09	1.18	8.99	2.08
	#213	-10.27	2.06	-7.22	-0.99	-9.27	-3.05
	#214	10.44	-2.87	7.13	0.44	9.99	3.31
	#215	12.80	-8.14	2.54	2.13	10.68	10.26
	#216	-11.87	13.45	2.10	-0.52	-11.35	-13.97
	#217	48.66	-16.84	13.44	15.55	36.70	28.81
	#218	-10.81	13.75	2.26	0.67	-11.49	-13.07
	#219	-38.64	47.98	6.53	2.81	-41.45	-45.17
	#220	-2.78	-16.24	-15.45	-3.58	0.80	12.66
	#221	-21.81	1.17	-12.93	-7.71	-14.10	-8.88
	#222	-17.07	-25.21	-37.10	-5.18	-11.89	20.03
	#223	-10.64	-5.62	-9.51	-6.75	-3.89	-1.13

Theoretically, DC current in AC grid due to two HVDC systems can be calculated by using the superposition principle. This means that DC currents in AC grid due to two HVDC lines with similar polarities will add up, while opposite polarity DC currents will cancel each other. The calculations results shown in Table 4 do match with this theoretical analysis. It can be observed for all three combinations of HVDC lines when two HVDC lines polarities are similar the DC currents are higher because of their additive effect. While if the polarities of two HVDC lines are opposite, the induced DC currents cancel each other thus reducing the net DC currents flowing in the AC grid. This concludes that two HVDC systems should be operated with opposite polarities monopole ground return modes to reduce the DC currents in AC grid.

#### Effect of AC substations connections on the DC currents

This subsection analyzes the effect of AC substation connections using the proposed circuital model technique. As a case study, different connection combinations of substation #10 are investigated when Lin-Feng HVDC system is operating under monopole ground return mode. Six different connection combinations of substation #10 with rest of the AC power grid are described in Table 5. The calculated DC currents in 500 kV and 220 kV substations are shown in Tables 6 and 7 respectively. Combination 0 in Table 5 shows the original connections where all the transmission lines are connected with substation #10. The DC current in substation #10 for the original connection combination is 30.57 A as can be seen in Table 6. The DC current, as per combination 4, in substations #10 reduced to 15.19 A when line 5195, 2101, 2102 and line 2113 are switched off. This combination reduces the DC current of substation #10 by 50.3%. The DC currents in other 500 kV substations are almost the same for different connection combinations of substation #10, as can be seen in Table 6. The DC current effect can be also analyzed for 220 kV substations by observing Table 7. The DC currents of substation #225 and #219 are reduced when the connection combination of substation #10 changes from 0 to 4. However, the DC currents of substation #221 and #223 increase for the aforementioned combination. Thus, we can conclude that the substation connection combination affects the DC currents in AC grid and various combinations can be analyzed for reducing the DC currents. The proposed technique can be effectively and efficiently used for performing such analysis because it is less computation intensive as compared to the conventional field-circuit coupled technique.

**Table 5:** Substation #10 connection combinations.

	Line 5195 to substation #14	Line 2101 to substation #223	Line 2102 to substation #223	Line 2113 to substation #218
Combination 0	on	on	on	on
Combination 1	off	on	on	on
Combination 2	off	off	on	on
Combination 3	off	off	off	on
Combination 4	off	off	off	off
Combination 5	on	off	off	off

**Table 6:** The DC currents in 500 kV substations under different connection combinations of Substation #10.

Substation	DC current (A)					
	Combination 0	Combination 1	Combination 2	Combination 3	Combination 4	Combination 5
#01	0.51	0.51	0.53	0.56	0.58	0.56
#02	-1.17	-1.15	-1.15	-1.14	-1.14	-1.17
#03	5.72	5.74	5.75	5.77	5.71	5.69
#04	9.84	9.84	9.84	9.85	9.88	9.89
#05	10.63	10.63	10.63	10.62	10.61	10.61
#06	1.34	1.38	1.41	1.47	1.42	1.36
#07	7.93	7.92	7.89	7.84	7.81	7.84
#08	5.81	5.68	5.61	5.48	5.62	5.82
#09	-0.69	-0.84	-0.75	-0.59	-0.84	-0.62
#10	-30.57	-29.37	-25.49	-17.86	-15.19	-18.11
#12	4.61	4.62	4.58	4.51	4.19	4.21
#13	10.84	10.93	11.09	11.38	11.12	10.96
#14	2.50	1.01	-0.43	-3.27	-3.51	-0.97

**Table 7:** The DC currents in part 220 kV substations under different connection combinations of Substation #10.

Substation	DC current (A)					
	Combination 0	Combination 1	Combination 2	Combination 3	Combination 4	Combination 5
#218	-12.28	-12.15	-11.74	-10.93	-10.62	-10.94
#219	-43.31	-43.15	-42.60	-41.51	-41.02	-41.42
#221	-11.49	-11.74	-12.58	-14.23	-15.18	-14.53
#223	-2.51	-2.49	-4.40	-8.14	-8.65	-8.15

#### Effect of substation location on the DC currents

The DC currents of terminal substation are usually more serious than those of other substations. At the terminal substation, only one transmission line is connected. As shown in Table 4, when Fu-Feng and Lin-Feng HVDC lines are operated with opposite polarities monopole ground return mode, the substations #202, #209 and #219 have very high DC currents. When Lin-Feng and San-Hu HVDC lines are operated with opposite polarities monopole ground return mode, the substations #209, #217 and #219 are with the highest DC currents. It can be noted from Fig 8 that all these substations are terminal substations. Such observations led us to suggest that new terminal substations location should be carefully designed. The problem of higher DC current can be reduced by selecting location of new terminal substation such that it lies on the equipotential lines with the existing substations.

## Conclusions

This work proposed and validated a novel circuital model methodology for DC currents calculation in AC grids which result due to HVDC system operating with monopole ground return mode. The proposed methodology can be applied to a complex power grid with any soil structure. The earth resistive network is obtained based on FEM simulations of the ground structure with HVDC grounding current injection. This earth resistive network is combined with AC resistive network and then solved for DC currents in AC grid. Our proposed methodology for the DC currents calculation is computationally more efficient as compared to the conventional field-circuit coupled techniques. The accuracy of the proposed methodology is proved by comparing the calculated DC current values with the actual measurements performed in two Shanghai area substations. Based on the DC current calculations using the proposed methodology, the DC currents mitigation measures in Shanghai area AC grid are suggested. It has been found that the DC current in AC system can be reduced by operating two HVDC systems with opposite polarities. Also, the AC grid connection combination and the location of new terminal substations can be designed for reducing the DC currents in AC grid.
